# RETRACTED: Wang et al. Panoramic Manifold Projection (Panoramap) for Single-Cell Data Dimensionality Reduction and Visualization. *Int. J. Mol. Sci.* 2022, *23*, 7775

**DOI:** 10.3390/ijms25053019

**Published:** 2024-03-05

**Authors:** IJMS Editorial Office

**Affiliations:** MDPI, St. Alban-Anlage 66, 4052 Basel, Switzerland; ijms@mdpi.com

The *Int. J. Mol. Sci*. Editorial Office journal retracts the article “Panoramic Manifold Projection (Panoramap) for Single-cell Data Dimensionality Reduction and Visualization” [1] cited above. 

Following publication, concerns were brought to the attention of the publisher by the originally listed authors, Yongjie Xu, Zelin Zang, Lirong Wu, and Ziqing Li, regarding the accuracy of the authorship, their permission to publish, and the authenticity of their email addresses as contained within this article [1].

Adhering to our complaints procedure, an investigation was conducted by the Editorial Office and Editorial Board that confirmed that the above mentioned persons (1) were unaware of the submission; (2) did not consent to be listed as authors; (3) were not responsible for the experimental results on the single-cell data as published; and (4) the email addresses, that were provided during the submission process, were not created or owned by the above mentioned persons.

As the appropriate authorship of this work cannot be established, nor could retroactive permission to publish the paper be obtained, the Editorial Office and Editorial Board have decided to retract this article as per MDPI’s retraction policy (https://www.mdpi.com/ethics#_bookmark29) and in line with the Committee on Publication Ethics retraction guidelines (https://publicationethics.org/retraction-guidelines). 

This retraction was approved by the Editor-in-Chief of the journal *Int. J. Mol. Sci*.

Yongjie Xu, Zelin Zang, Lirong Wu, and Ziqing Li agreed to this retraction and Yajuan Wang did not agree to this retraction.
